# Autologous Fat Graft Combined With Botulinum Toxin Injection for Breast Augmentation in Poland Syndrome: A Prospective and Comparative Study

**DOI:** 10.1111/jocd.70070

**Published:** 2025-02-14

**Authors:** Na Wang, Siming Wei, Shuai Qiang, Juan Wang, Xianhui Zeng, Zhaoxiang Zhang

**Affiliations:** ^1^ Department of Plastic and Reconstructive Surgery, Xijing Hospital Forth Military Medical University Xi'an Shaanxi China

**Keywords:** Augmentation, Autologous fat, Botulinum toxin

## Abstract

**Background:**

Poland syndrome is a rare congenital condition characterized by unilateral breast deformity. Autologous fat transplantation has emerged as the preferred treatment due to its minimal invasiveness, rapid recovery, absence of rejection reactions, and potential for multiple surgeries to enhance postoperative outcomes. Previous animal studies have shown that botulinum toxin significantly improves fat retention rates following fat transplantation. Therefore, we aim to initiate a clinical study to investigate the effects of botulinum toxin on human fat transplantation.

**Objective:**

This prospective comparative clinical study aims to evaluate the impact of combining botulinum toxin with autologous fat grafting on fat retention rates in patients with Poland syndrome.

**Method:**

From October 2017 to December 2023, we enrolled 20 Poland syndrome patients, assigning them to an experimental group receiving fat and botulinum toxin for breast augmentation and a control group undergoing standard autologous fat grafting. Postoperative fat retention rates were compared, and outcomes were assessed using the Breast‐Q score, alongside baseline patient data.

**Results:**

There were no significant differences in baseline data between the two groups. At 3 and 6 months postoperatively, the fat retention rate in the experimental group was significantly higher than that in the control group. Regarding Breast‐Q scores, the control group exhibited significantly lower scores in the Satisfaction with breast domain than the experimental group, with no notable differences in other domains.

**Conclusion:**

The injection of a mixture of fat and botulinum toxin significantly enhances fat retention rates in patients with isolated breast deformities associated with Poland syndrome.

**Trial Registration:**

This study has been registered with the China Clinical Trial Center (ChiCTR2100054878)

## 1 Introduction

Poland syndrome is a rare genetic disorder characterized by a variety of musculoskeletal abnormalities affecting one side of the breast and upper limb [1]. The majority of patients with Poland syndrome primarily present with breast deformities resulting from the absence of the pectoralis major muscle in the chest wall. Less common manifestations may include rib deformities, hypoplasia of the breast and nipple, ectopic or absent axillary hair, syndactyly or brachydactyly of the fingers, and mild shortening of the forearm bones or underdevelopment of the muscles, among others [2, 3]. A very small number of patients may also present with congenital defects outside the musculoskeletal system, such as pulmonary malformations, renal malformations, and dextrocardia [4]. Therefore, the primary focus of orthopedic treatment for most patients is to address breast deformities. For many individuals with Poland syndrome, unilateral breast deformity or asymmetry may appear straightforward. However, such unilateral defects can substantially affect overall appearance, which may, in turn, influence the patient's social relationships and contribute to various psychological issues [5].

The patients' desire is to address breast asymmetry through the simplest surgical intervention possible, minimizing additional trauma, in order to achieve a normal appearance and aesthetic outcome [6]. In this context, autologous fat grafting has become the preferred treatment for patients with Poland syndrome [1]; however, the instability of fat retention remains an unavoidable issue in fat transplantation. Our previous animal experimental research indicated that the application of fat grafting combined with botulinum toxin can promote the proliferation of adipose‐derived stem cells in mice and enhance the density of neoangiogenesis, thereby improving the retention rate of transplanted fat [7, 8]. Consequently, we aim to conduct a prospective and comparative clinical study to investigate whether botulinum toxin similarly improves fat retention rates in Poland syndrome patients undergoing fat grafting.

## 2 Patients and Methods

This study adheres to the Helsinki Declaration, has received approval from the Ethics Committee and has been registered with the China Clinical Trial Center. Inclusion criteria are as follows: (1) aged 16–45 years; (2) female patients diagnosed with Poland syndrome characterized by unilateral absence of the pectoral muscle with incomplete breast development; (3) intact upper limb function; and (4) signed informed consent. Exclusion criteria are as follows: (1) severe chest wall deformities or skeletal defects; (2) Previous major breast surgery that may impact the prognosis of fat transplantation; (3) presence of other conditions unsuitable for autologous fat grafting; and (4) withdrawal from the study midway.

### 2.1 Patients Enrollment

From October 2017 to December 2023, a total of 20 female patients with Poland syndrome were recruited. Using a random number table, we evenly divided the participants into an experimental group and a control group. The control group received autologous fat grafting alone for breast augmentation, while in the experimental group, the fat was mixed with botulinum toxin prior to injection for breast augmentation.

### 2.2 Surgical Technique

**FIGURE 1:**
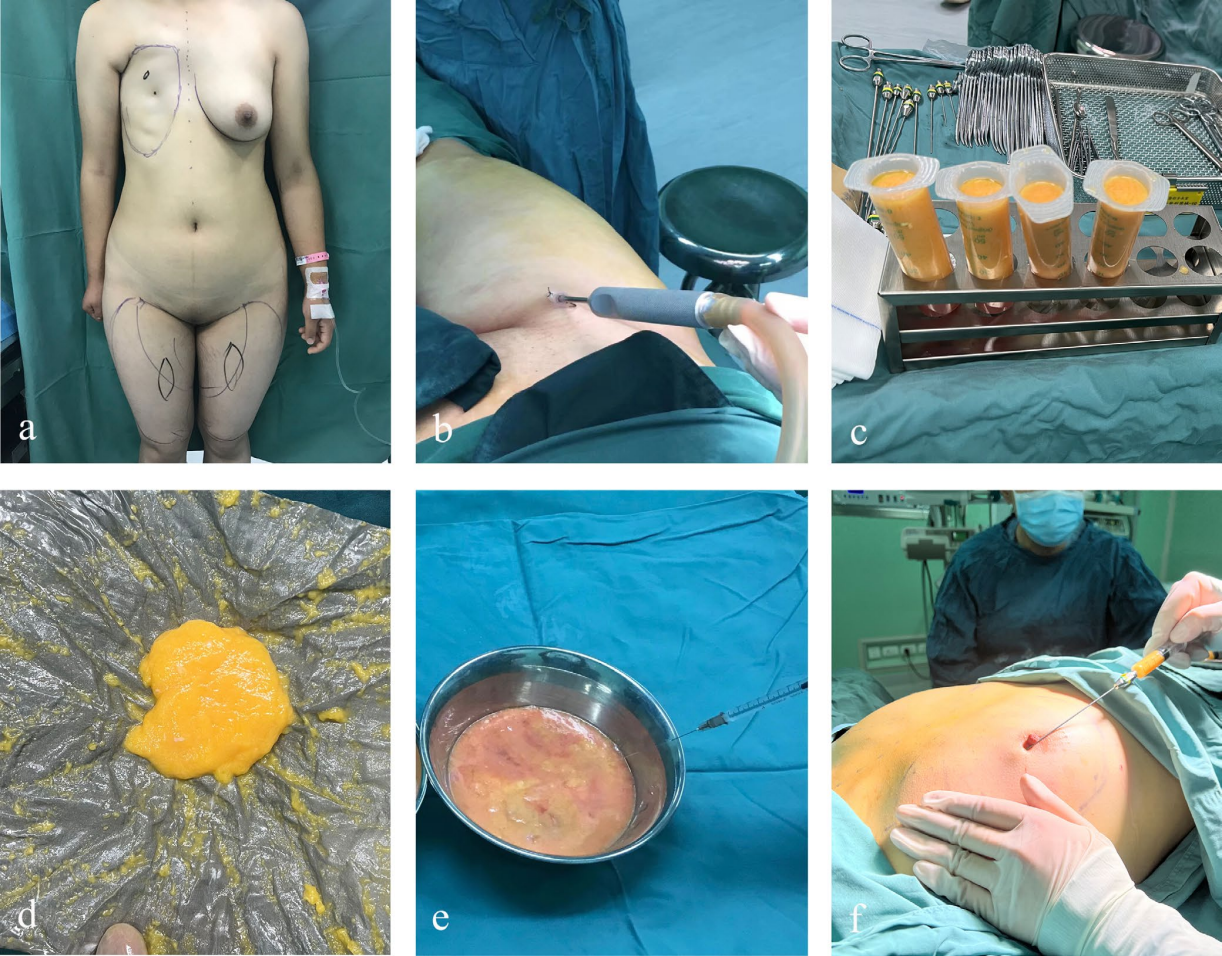
(a) Preoperative design; (b) fat extraction; (c) fat centrifugation; (d) fat filtration; (e) botulinum mixing; and (f) fat injection.

Prior to surgery, all patients underwent evaluation of the required volume of fat to be transplanted using a 3D camera. Considering future pregnancy demands among some patients, we chose the inner thigh as the donor area for fat grafting to avoid adverse effects on abdominal expansion caused by subcutaneous scars and trauma. To minimize potential errors, the steps of fat collection, processing, and injection were performed by the same surgeon using the Coleman technique. Before liposuction, we first marked the donor area with a pen to indicate the peak of the fat transfer and the volume and range of fat required by the patient. During the procedure, the patient was positioned supine, and the surgical area was disinfected with povidone‐iodine. A tumescent solution was prepared using 1 L of Hartmann's solution, 2 mL of epinephrine (1 mg/1 mL), and 10 mL of lidocaine (5 g/L) for local anesthesia. The tumescent solution was then injected into the donor area to facilitate fat extraction and minimize trauma. The ratio of harvested fat to tumescent solution was approximately 1:1. After liposuction, the fat was centrifuged using the Sterile Squeeze Centrifugal Lipotransfer (SCL) system at 2000 r/min for 3 min to enhance the density of adipose‐derived stem cells (ASCs) and mesenchymal structures by removing old fat cells and liquid triglycerides. During centrifugation, a weighted mesh filter bottle was used to compress and aspirate the fat in the same syringe, breaking down larger and older fragile fat cells, concentrating the fat tissue while removing liquid triglycerides and impurities. In the experimental group, the centrifuged fat was additionally mixed with botulinum toxin at a ratio of 100 mL of fat to 100 U of botulinum toxin A (Lanzhou Hengli), prepared with 2.5 mL of saline. For fat injection, we selected the inframammary fold and the periareolar region as incision sites, with incisions approximately 3 mm in length. A 17‐gauge blunt needle was used to connect the filled syringe, allowing for multi‐channel and multi‐plane injections of fat into the subcutaneous tissue while avoiding entry into the intercostal space. Finally, the incisions were sutured with 7–0 nylon thread. Postoperatively, patients were advised to refrain from excessive use of the right arm for two weeks, and we did not employ the massage techniques typically used for silicone implants. To prevent excessive fat absorption or liquefaction from a single large graft, we scheduled at least two procedures for each patient, with a minimum interval of six months between surgeries. Subsequent surgeries would be arranged only until the total fat retention rate reached approximately 80%, at which point treatment would be considered complete (Figure 1a–f).

### 2.3 Assessment Item

**FIGURE 2:**
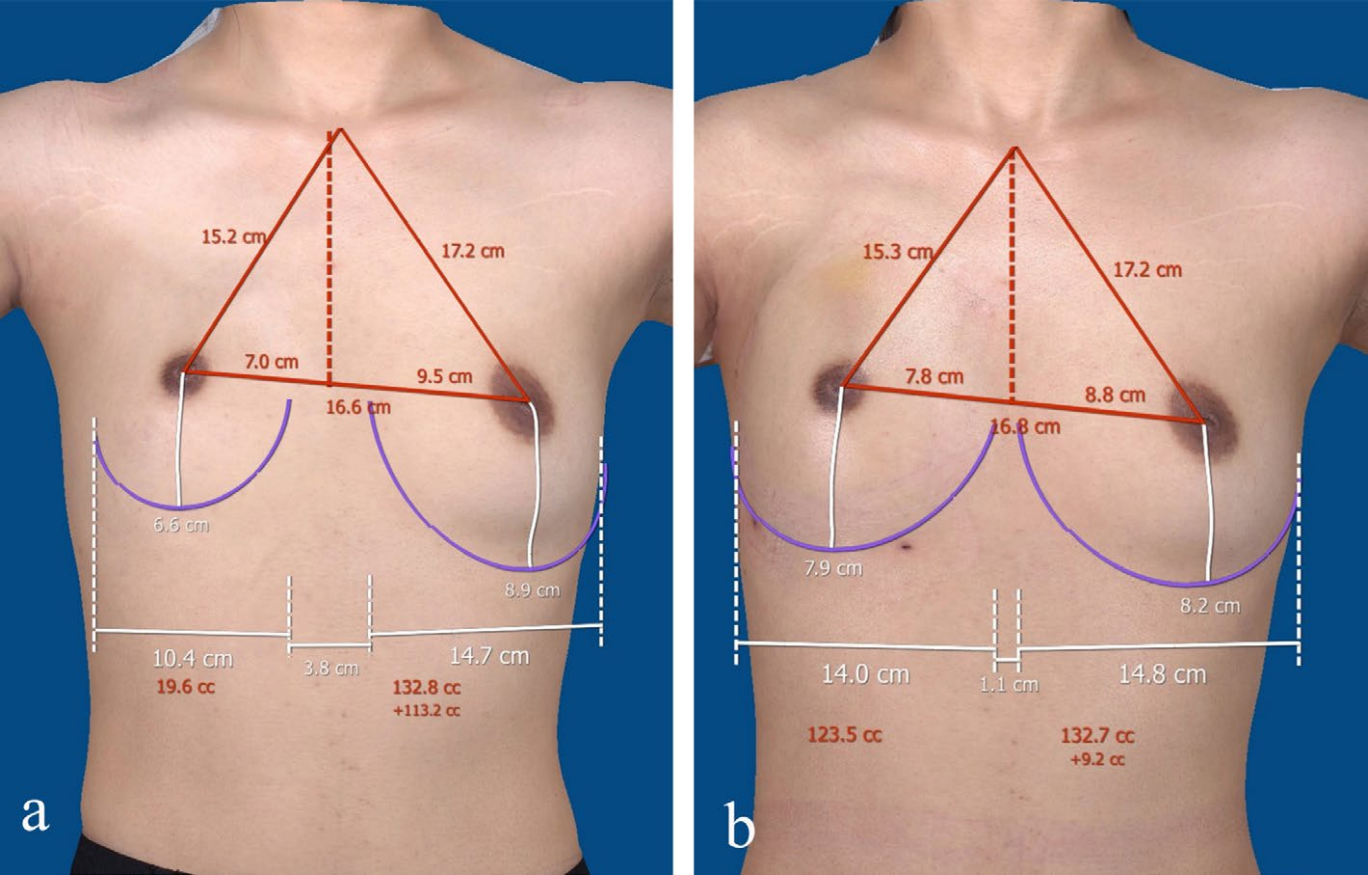
3D photos of control group patients. (a) Preoperative 3D photo (showing a 113.2 cc difference in fat volume between the patient's two sides) and (b) 3D photos two years postoperatively (showing a 9.2 mL difference in fat volume between the two sides).

The indicators we need to collect include the following: (1) baseline data of the patients, such as age, body mass index (BMI), and difference in bilateral breast volume; (2) a comparison of the number of surgeries experienced by both groups of patients when the fat retention rate reached approximately 80%; (3) fat retention rates of both groups at 3 and 6 months postoperatively (to minimize errors due to differing numbers of surgeries among patients, we measured only the fat retention rate after the first surgery). We utilized a 3D camera (Vectra XT, Canfield Corp., CA, USA) to calculate fat retention rates (Figure 2); (4) the Breast‐Q scores [9] of both groups at 6 months postoperative. This scale is designed for surgical interventions in the breast domain and includes five modules: Breast Augmentation, Reduction Mammaplasty, Mastectomy, Breast‐Conserving Therapy, and Breast Reconstruction. Each module primarily assesses patient satisfaction, physical health, and sexual health, with individual scores ranging from 0 to 100, where higher scores indicate greater patient satisfaction or quality; and (5) a record of postoperative complications.

### 2.4 Statistical Analysis

We analyzed the data using SPSS 26.0 (IBM Statistics for Windows, IBM Corp., Armonk, NY, USA). For each dataset from the two patient groups, we represented the data using the median. Considering the non‐normal distribution characteristics of the patient data in this study, we employed the Mann–Whitney *U* test to analyze the differences. A *p* value < 0.05 was considered statistically significant.

## 3 Results

The experimental group comprised 10 patients, with a median age of 30.00 years. The median BMI was 19.38 kg/m^2^, and the difference in bilateral breast volume was 167.50 mL. In comparison, the control group had a median age of 25.50 years, a BMI of 21.02 kg/m^2^, and a difference in bilateral breast volume of 157.50 mL. There were no significant differences in baseline characteristics between the two groups (Table 1).

**TABLE 1:** Patient baseline data.

Item	Experimental group	Control group	*p*
Age (years)	30.00 (27.25, 37.25)	25.50 (25.50, 36.50)	0.790
BMI (kg/m^2^)	19.38 (18.20, 21.93)	21.02 (19.65, 22.19)	0.226
Difference in bilateral breast volume (mL)	167.50 (125.25, 212.75)	157.50 (124.00, 221.75)	0.821

*Note:* All data are presented using the median (P25, P75) and analyzed with the Mann–Whitney *U* test.

The fat retention rates in the experimental and control groups were 55.12% and 47.54%, respectively, at 3 months after the first surgery. At 6 months post‐operation, these rates were 43.67% and 35.64%, respectively. The fat retention rates in the experimental group were significantly higher than those in the control group, with this difference becoming more pronounced at 6 months compared to 3 months post‐operation. The median number of surgeries for achieving approximately 80% fat retention was 3 in both the experimental and control groups, with no significant difference between the two (Table 2).

**TABLE 2:** Comparison of surgical frequency, fat retention rate, and Breast‐Q scores between the two groups.

Item	Experimental group	Control group	*p*
Operation frequency	3 (2, 4)	3 (2, 4)	0.240
Fat retention rate			
Postoperative 3 m%	55.12 (52.36,57.49)	47.54 (43.80,48.96)	< 0.01*
Postoperative 6 m%	43.67 (40.85,46.67)	35.64 (31.44,38.21)	< 0.01*
Breast‐Q			
Satisfaction with breast	80.00 (71.50,85.00)	65.00 (60.75,69.75)	0.01*
Satisfaction with outcome	82.00 (72.25,92.50)	76.50 (72.25,87.00)	0.60
Psychosocial well‐being	81.50 (77.75,85.50)	75.50 (66.00,86.25)	0.21
Sexual well‐being	76.50 (63.50,83.25)	72.50 (67.75,81.25)	0.82
Physical well‐being	87.00 (75.00,89.50)	83.00 (77.75,89.00)	0.97
Satisfaction with information	87.50 (82.50,90.50)	85.00 (83.00,89.00)	0.52

*Note:* All data are presented using the median (P25, P75) and analyzed with the Mann–Whitney *U* test.

* The mean difference is significant at the 0.05 level.

There were no significant differences between the two groups of patients in terms of Satisfaction with outcome, psychosocial well‐being, physical well‐being, sexual well‐being, and satisfaction with information. However, in terms of Satisfaction with breast, patients in the experimental group gave significantly higher ratings compared to those in the control group (Table 2).

Regarding postoperative complications (Table 3), the most common complication observed was the formation of calcified nodules, occurring in 3 patients from the experimental group and four patients from the control group. A small number of patients also experienced hematomas. Two weeks post‐operation, one patient in the control group developed an incision infection, which was promptly resolved after disinfection and dressing changes, likely due to improper postoperative care. No patients experienced serious complications such as fat embolism.

**TABLE 3:** Postoperative complications statistics.

Complications	Experimental group	Control group
Hematoma	2	1
Calcified nodule	3	4
Infection	0	1
Fat embolism	0	0

## 4 Cases Report

### 4.1 Case 1

**FIGURE 3:**
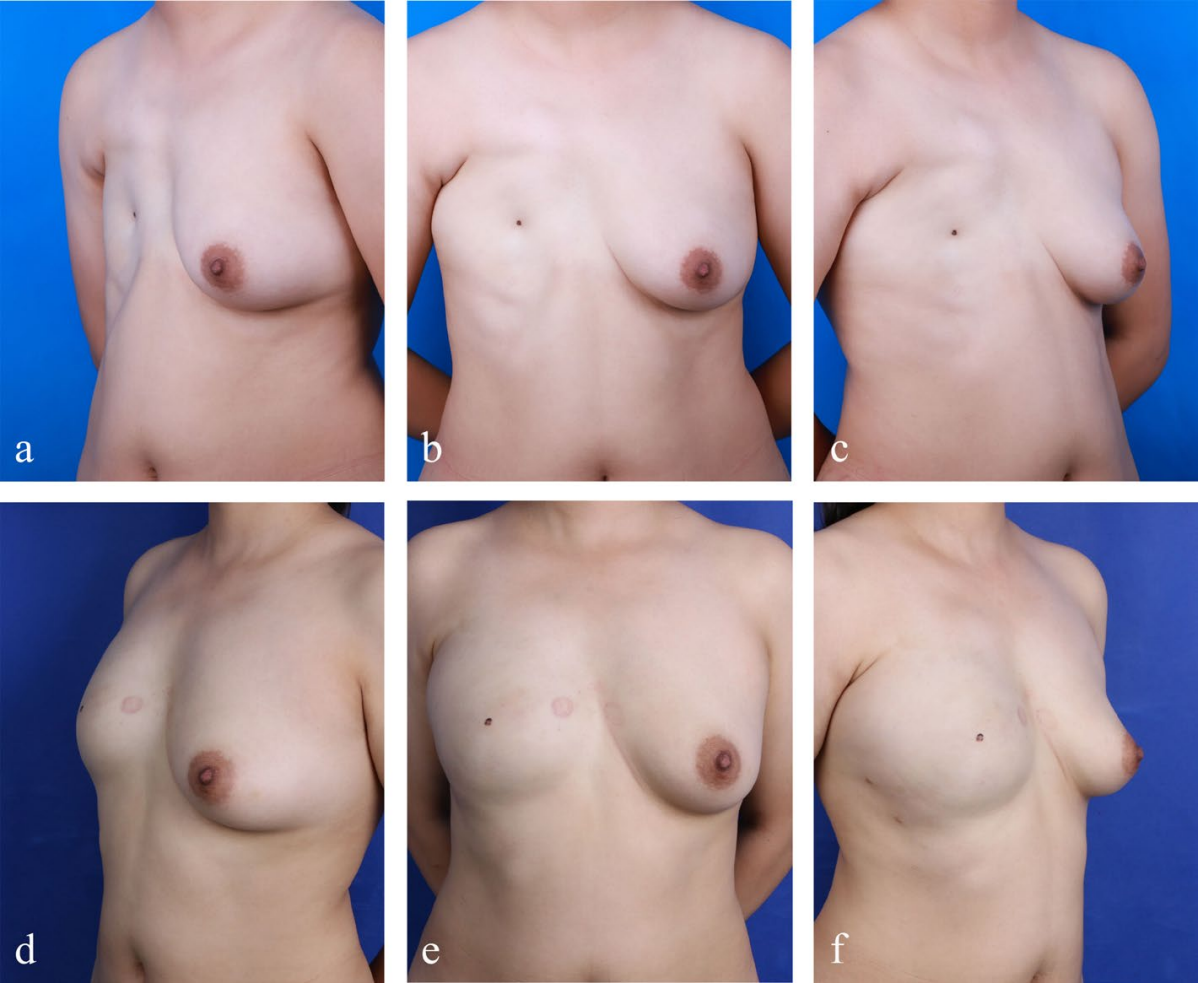
Treatment Process of Experimental Group Patients. (a–c) Preoperative photos of the experimental group patients and (d–f) stable phase photos of experimental group patients after treatment.

The female patient in the experimental group was 26 years old and presented with a congenital right breast deformity, diagnosed as Poland syndrome upon admission in 2019. Given the substantial trauma associated with flap reconstruction surgery and the associated risks of prognosis, the patient opted to decline breast reconstruction surgery. Instead, she preferred to pursue autologous fat grafting to achieve a better breast appearance. Preoperatively, we estimated that approximately 280 mL of fat would be needed to achieve adequate filling of the left breast. A total of three surgeries were performed, during which 500 mL of fat was transplanted, mixed with 500 U of botulinum toxin. The fat retention rates were 56% and 44% at 3 and 6 months post‐operation, respectively, and the Breast‐Q scores were notably high. The patient was extremely satisfied with the postoperative results, achieving her preoperative goals and fully resuming her normal life (Figure 3a–f).

### 4.2 Case 2

**FIGURE 4:**
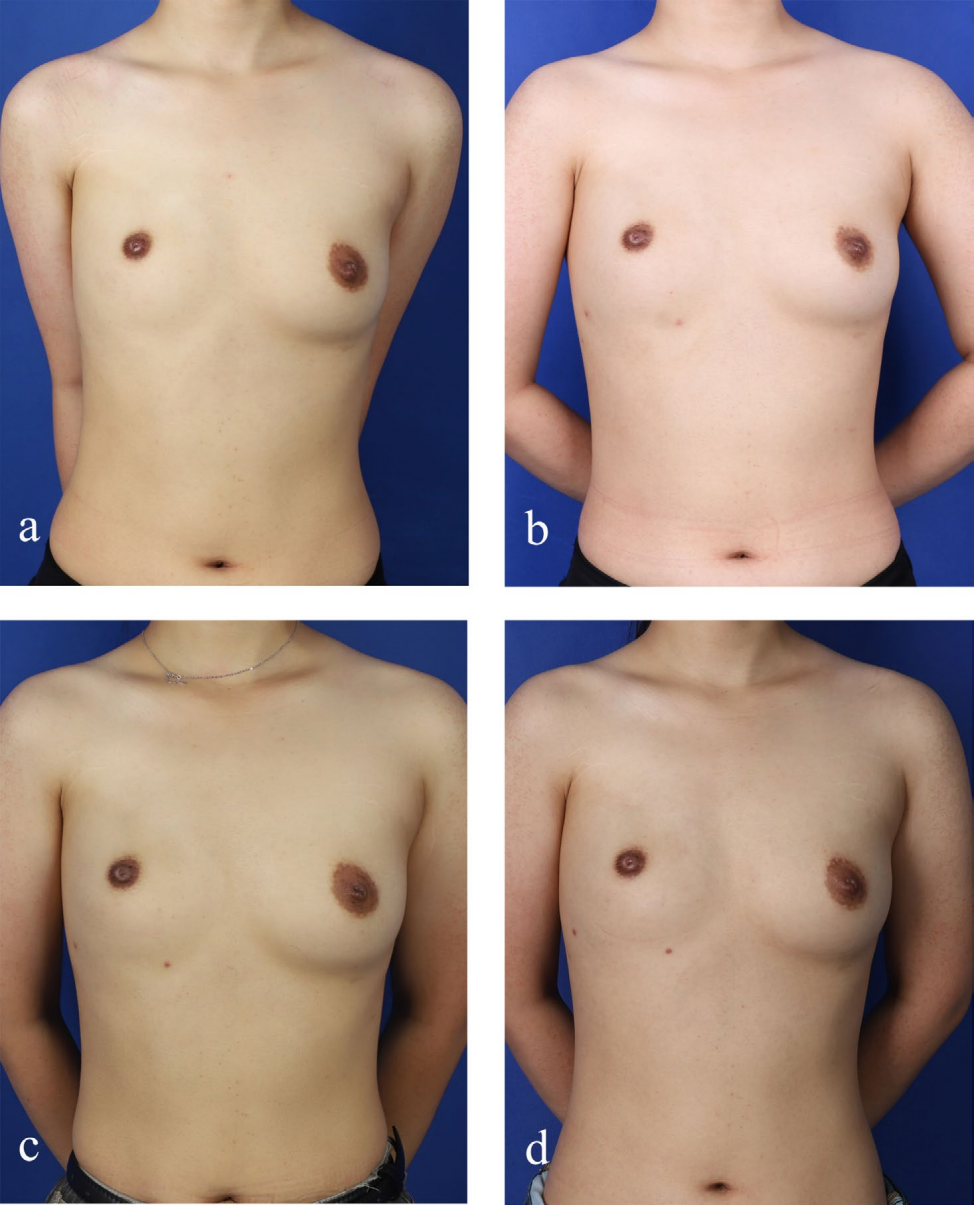
Treatment process of control group patients. (a) Preoperative photo of the control group patient; (b) photo of the control group patient at 3 months post initial surgery; (c) photo of the control group patient at 6 months post initial surgery; (d) stable phase photos of control group patients after treatment.

The 22‐year‐old female patient in the control group was admitted in 2022 and diagnosed with Poland syndrome, characterized by congenital absence of the right pectoralis major muscle with nipple‐areola malformation. Her left pectoralis major muscle developed normally without chest wall deformities, and she had good upper limb function. Like the patient in the experimental group, her primary concern was restoring a normal appearance to her breast with minimal surgical intervention, leading to the choice of autologous fat transplantation. She underwent a total of three surgeries, receiving a total of 300 mL of fat transplanted. Fat retention rates were 45% and 36% at 3 and 6 months post‐operation, respectively, with high Breast‐Q scores. The initial postoperative fat retention rate was not ideal; however, the patients expressed satisfaction with the final surgical outcomes. (Figure 4a–d).

## 5 Discussion

Poland syndrome represents a rare congenital disorder characterized by unilateral breast and upper limb anomalies, with potential multiorgan involvement including cardiac, pulmonary, renal, vertebral, and coagulation system abnormalities. First documented by Lallemand (1826) and subsequently elucidated through Alfred Poland's 1841 autopsy report of a 27‐year‐old male criminal at Guy's Hospital, the condition was formally designated “Poland syndrome” by Patrick Clarkson in the 20th century [10]. Epidemiological data indicate a prevalence of 1:30000 with male predominance (3:1 ratio) and right‐sided laterality preference (3:1 ratio) [11, 12]. The predominant etiological hypothesis involves embryonic vascular disruption of subclavian and vertebral artery branches around gestational week 6, conceptualized as the Subclavian Artery Supply Disruption Sequence (SASDS) [13, 14]. This mechanism may share pathogenic pathways with Moebius syndrome, Klippel‐Feil syndrome, and Adams‐Oliver syndrome [15, 16].

Clinical presentations in plastic surgery practice typically involve mild functional impairment but significant aesthetic concerns, particularly regarding breast contour restoration [17]. Therefore, their main concern is addressing aesthetic issues, restoring a normal breast contour, and returning to normal social life. Conventional reconstructive approaches, including pedicled/flap surgeries, present limitations: latissimus dorsi flaps demonstrate inadequate volume persistence [18], while transverse rectus abdominis flaps offer ample autologous tissue; complications at the donor site (such as hernias and weakened abdominal walls) limit their use [19], while implants face restrictions in lean patients and higher complication rates [20, 21]. In contrast, autologous fat transplantation emerges as a superior option due to minimal invasiveness, absence of foreign body reactions, and cost‐effectiveness.

Optimizing fat retention rates remains challenging, largely due to vascularization efficiency and the differentiation of adipose‐derived stem cells (ADSCs) [22]. Our previous mouse experiments showed that botulinum toxin inhibits muscle activity while enhancing neovascular density and mature adipocyte density. This improves the histological characteristics of transplanted fat and increases fat retention rates. Previous research has indicated that BTX‐A promotes angiogenesis via the hypoxia‐inducible factor‐1α/vascular endothelial growth factor pathway, improving the survival rate of rectus abdominis myocutaneous flaps [23]. In another study, BTX‐A was injected into the center of the flap, leading to a significantly higher survival rate in the BTX‐A group. The researchers observed larger vascular diameters and a greater number of immature blood vessels [24]. This effect may be linked to botulinum toxin's ability to reduce norepinephrine secretion [25]. Studies have shown that botulinum toxin improves perfusion and stimulates adipose stem cell proliferation [26]. It also influences key signaling molecules, such as the mitogen‐activated protein kinase (MAPK) pathway, which plays a crucial role in cellular processes like proliferation and differentiation, promoting adipose stem cell differentiation into adipocytes [27]. Additionally, botulinum toxin alters the cellular microenvironment by regulating cytokine secretion from surrounding cells, including insulin‐like growth factor (IGF) [28]. IGF plays a significant role in promoting the adipogenic differentiation of adipose stem cells. Based on these studies, we will conduct clinical research to validate botulinum toxin's role in improving human fat transplantation survival rates, aiming to confirm our hypothesis. In this study, we hypothesize that botulinum toxin enhances fat retention by: (1) increasing neovascular density and (2) promoting adipose stem cell proliferation. However, further basic research is needed to verify these effects. Since most patients in this study lacked pectoral muscles, further research is needed to confirm botulinum toxin's role in stabilizing fat by immobilizing muscles. Several studies have explored the effect of botulinum toxin on fat survival rates. Chun‐Shin Chang and colleagues reported favorable treatment outcomes using botulinum toxin to assist facial fat grafting [29]. Gugerell and colleagues found that lower botulinum toxin concentrations promoted cell proliferation, while higher concentrations induced apoptosis [30]. Wang and colleagues summarized botulinum toxin's applications in fat grafting and other plastic surgeries, highlighting its potential to enhance fat survival rates through various mechanisms. Our previous research also demonstrated the effectiveness of botulinum toxin in enhancing fat retention during breast augmentation [31].

The male‐to‐female incidence ratio of Poland syndrome is approximately 3:1. This is due to recruited patients primarily presenting with pectoral muscle absence and breast deformities, without other skeletal or functional impairments. Patient concerns were mainly focused on correcting breast appearance, leading many male patients to perceive no need for treatment, as the condition did not significantly affect daily life. Consequently, the majority of patients seeking treatment and enrolling in our study were female. The follow‐up period was set at 3 and 6 months post‐surgery. Due to the need for balanced baseline data and multiple fat grafting procedures, we cannot extend the follow‐up period beyond 6 months. Fat survival rates show the most significant changes within the first 6 months; thus, we focused our follow‐up on this period. Our study's success criterion was achieving a fat retention rate of around 80%, eliminating the need for further fat transplantation surgeries. We also analyzed the number of surgeries each group underwent, showing no significant difference between the two groups (*p* = 0.240). With more participants in the study group, we expect significant differences in surgical frequency between the two groups, based on the superior fat retention rates observed per surgery. Regarding the Breast‐Q scale, apart from the Satisfaction with Breast indicator, there were no significant differences in patient evaluations between the two groups. Satisfaction with breast scores were significantly lower in the control group compared to the experimental group. These patients may perceive their restored breast contours as fragile. However, we believe that with time, these patients will gradually overcome these sensitivities and anxieties.

Our study has several limitations. First, the sample size is small due to the rarity of Poland syndrome. Future multicenter studies with larger sample sizes are needed to confirm these findings. Second, this study only assessed fat survival at 6 months post‐surgery. Future research should include longer follow‐up periods and pathological analysis of fat samples. Third, Poland syndrome has a male‐to‐female incidence ratio of 3:1. However, women generally have a greater demand for favorable breast appearance, potentially leading to gender selection bias in patient inclusion. We will further explore the impact of gender differences by including additional cases. Finally, while we have explored the biological mechanisms of botulinum toxin's effect on fat retention through animal experiments, further in‐depth human studies are needed.

## 6 Conclusion

We conducted a prospective comparative clinical study on patients with Poland syndrome and isolated breast deformities. Injecting a mixture of fat and botulinum toxin significantly improved fat retention rates and led to satisfactory postoperative outcomes.

## Author Contributions

N.W., S.W., and S.Q. performed the research. Z.Z. supervised the research study. S.Q., J.W., and X.Z. analysed the data. N.W. and S.W.wrote the paper. N.W., S.W., and Z.Z. performed the operation.

## Ethics Statement

This study has received approval from the Ethics Committee of Xijing Hospital.

## Conflicts of Interest

The authors declare no conflicts of interest.

## Data Availability

The data that support the findings of this study are available from the corresponding author upon reasonable request.
